# The effect of pH and transition metal ions on cysteine-assisted gold aggregation for a distinct colorimetric response

**DOI:** 10.1039/d1ra90091a

**Published:** 2021-03-17

**Authors:** Trang Thi Thuy Nguyen, Olivia A. Han, Eun Bi Lim, Seungjoo Haam, Joon-Seo Park, Sang-Wha Lee

**Affiliations:** Department of Chemical and Biological Engineering, Gachon University 1342 Seongnamdaero, Sujeong-gu Seongnam-si Gyeonggi-do 13120 Republic of Korea lswha@gachon.ac.kr; Department of Chemistry, Eastern University 1300 Eagle Road, St. Davids PA 19087-3696 USA jpark6@eastern.edu; Department of Chemical and Biomolecular Engineering, Yonsei University 50 Yonsei-ro, Seodaemun-gu Seoul 03722 Republic of Korea

## Abstract

Correction for ‘The effect of pH and transition metal ions on cysteine-assisted gold aggregation for a distinct colorimetric response’ by Trang Thi Thuy Nguyen *et al., RSC Adv.*, 2021, **11**, 9664–9674, DOI: 10.1039/D1RA00013F.

The authors regret that in the original article an incorrect email address was given for the corresponding author Sang-Wha Lee. The correct email address is shown here.

The Royal Society of Chemistry apologises for these errors and any consequent inconvenience to authors and readers.

## Supplementary Material

